# Supplementary material for: FedGMMAT: Federated generalized linear mixed model association tests
Source: PLoS Comput Biol. 2024 Jul 24;20(7):e1012142. doi: 10.1371/journal.pcbi.1012142 (PMC11299833; doi:10.1371/journal.pcbi.1012142)
Supplement: S1 Text — (PDF) [file pcbi.1012142.s001.pdf]

Supplementary Information for "FedGMMAT: Federated Generalized Linear Mixed Model Association Tests"

1 Appendices

1.1 Impact of Kinship Heterogeneity on GWAS Results

We provide a motivational example of how kinship heterogeneity among case and control cohorts may impact GWAS analysis results unless they are accounted for using mixed effect models.

To demonstrate the importance of modeling the random effects confounded by kinship, we performed tests where we ran GMMAT and plink2 (non-mixed effects model method) on simulated datasets with high levels of heterogeneity in relatedness among the case/control cohorts. We first simulated genotype data of 6500 variants for 4800 subjects from the GBR population in The 1000 Genomes Project. Among these, 2400 subjects are simulated as outbred individuals. Remaining 2400 subjects are simulated from 100 pedigrees of 24 subjects with moderate-to-high levels of inbreeding. To evaluate the biases, the phenotypes were simulated with no genetic component, i.e., the phenotypes have no genetic component (zero heritability): The inbred cohort is specifically set as the case cohort as the subjects with disease and the outbred cohort is set as the control cohort. For each subject, 5 PCs and a randomly assigned gender was used as fixed covariates.

We performed GWAS analysis to calculate the association p-values for the 6500 variants using plink2 and GMMAT using the simulated dataset. Specifically, GMMAT was tested with kinship matrix inferred using SIGFRIED. As a negative control, we tested the GMMAT with a trivial kinship matrix, i.e., diagonal kinship matrix to simulate the lack of kinship information. We observed that there is strong inflation of p-values in plink2's p-values (S1 Fig). As expected, GMMAT exhibits the same inflation of the p-values when it is run with the trivial (i.e., diagonal) kinship matrix. When GMMAT is used with the inferred kinship matrix, we observed that the kinship-based biases p-values are appropriately corrected by GMMAT.

Overall, this result demonstrates an example scenario where the kinship heterogeneity among cases and controls may bias the results when cryptic kinship exists more in the cases or controls by random chance. We do acknowledge that this is a severe case of cryptic relatedness, and it is aimed at demonstrating the importance of considering polygenic effects caused by kinship. We also acknowledge that there can be different cases where cryptic kinship can bias the results [1].

1.2 Data Aggregation Protocol

FedGMMAT relies on secure data aggregation and partitioning protocols (S2 Fig). These protocols that make use of following steps:

(1) encrypting data locally at each site: Each site use the agreed upon encryption scheme to encrypt data. Before this, the sites add site-specific partitioned noise, $N^{(part)_{(j)}}$, to protect their own share of the sensitive

matrix data. After adding the partitioned noise matrix, the sites encrypt the matrix using Homomorphic encryption key (or OTP-like encryption using the secret key matrix generated using the key from server).

(2) pooling the data using round-robin schedule: Data is aggregated among all sites. Round robin schedule is used for this purpose by default. More efficient approaches can be implemented to make this step faster.

(3) Decrypting the aggregated data at the central server: Aggregated and encrypted data is sent to server, where it is decrypted using HE private key or the OTP-like key matrix (S3 Fig). The decrypted matrix contains only the partitioned noise matrix from all sites.

(4) Partitioning the data and sending the corresponding partition back to each site. Each site removes their partitioned noise component and obtain the plaintext aggregated matrix partition to be used for analysis.

1.3 Quasi-Likelihood (QL)

With the preparation of the initial fixed covariate coefficient $\tilde{\boldsymbol{\alpha}}_0$ is done, we are ready to estimate the mixed-effect $\boldsymbol{b}$. Reminds that $\boldsymbol{b}$ follows a distribution $\mathcal{N}(0, \tau\mathbf{V})$, and with a little abuse of notation, we denote the linear part $\mu_{ij} = \mathbf{X}_{ij}\boldsymbol{\alpha} + G_{ij}\beta + b_{ij}$, the probability of binary outcome $\pi_{ij} = g(\mu_{ij}) = P(y_{ij} = 1|\mathbf{X}_{ij}, G_{ij}, b_{ij})$, a variance function $\nu(\cdot)$, and $Var(y|\boldsymbol{b}) = \nu(\mu)$. Then, the log-quasi-likelihood function is of form

$$\begin{aligned} ql(\boldsymbol{\alpha}, \beta, \tau) = \log \int \exp &\left\{ \sum_{j=1}^{J} \sum_{i=1}^{n_j} ql_{ij}(\boldsymbol{\alpha}, \beta, \boldsymbol{b}; y_{ij}) \right\} \\ &\times (2\pi)^{n/2} |\tau V|^{-1/2} \\ &\times \exp\left\{ -\frac{1}{2} \boldsymbol{b}^\top (\tau V)^{-1} \boldsymbol{b} \right\} d\boldsymbol{b} \end{aligned} \tag{1}$$

where

$$ql_{ij}(\boldsymbol{\alpha}, \beta, \boldsymbol{b}; y_{ij}) = \int_{y_{ij}=1}^{\mu_{ij}} \frac{y_{ij} - \mu_{ij}}{\nu(\mu)} d\mu$$

1.4 Pearson Chi-square approximation

$$\sum_{j=1}^{J} \sum_{i=1}^{n_j} ql_{ij}(\boldsymbol{\alpha}, \beta, \boldsymbol{b}; y_{ij}) = \sum_{j=1}^{J} \sum_{i=1}^{n_j} \int_{y_{ij}=1}^{\mu_{ij}} \frac{y_{ij} - \mu_{ij}}{\nu(\mu)} d\mu \tag{2}$$

$$\approx \sum_{j=1}^{J} \sum_{i=1}^{n_j} \frac{(y_{ij} - \mu_{ij})^2}{\nu(\mu_{ij})} \tag{3}$$

2 Extensive Discussion of Limitations of FedGMMAT

We list below a general list of limitations and criticisms of FedGMMAT that warrant further research in later studies:

1. Lack of Random Slopes: As we discussed above, a random slopes model can help further characterize and control confounders. This must be formulated carefully to ensure that null model is not overly relaxed,

which may affect model's power to detect associations.

2. Design Constraints in Federated GWAS: The sites must have designed the study with same covariate information and same study design. One of the criticisms (rather than limitations) to FedGMMAT and other federated algorithms is that most studies designed this way would have an accompanying data sharing and analysis plan and can potentially share data among sites rather than performing a pooled analysis. However, this may cause concerns among participants and decrease sample sizes. We agree that this is . The studies that would benefit from the usage of federated algorithms may therefore be limited due to challenges of finding similar study designs.

3. Requirements of a Federated PCA: The sites must execute a federated PCA to obtain the most accurate set of population confounding PC covariates. However, the federated PCA approaches in the literature are highly computationally demanding and may fail to correctly identify confounders when there are related individuals in the cohorts. To get around these issues, the sites can use a projection-based approach that we proposed in a previous study, which would also be effective to remove a large portion of the confounding. This approach is also very computation efficient (one projection only) and is performed only locally. The limitation of this approach is the reliance on an existing large cohort, we foresee that the existence of very large cohorts such as AllofUS and UKBioBank help for this purpose. Numerous studies in the literature have pointed out the advantages of using projection-based approaches for federated studies and meta-analysis to partially mitigate biases. We believe that new reference databases can help build comprehensive projection-based approaches.

4. Comparison with Meta-Analysis: The potential of usage of meta-analysis approaches, which already provide most strongly associated variants in association tests, are already dominant mode of analysis in performing multi-site and retrospective analysis. However, these advantages also reflect back as potential limitations: Numerous studies demonstrated that the meta-analysis may bias results when there is heterogeneity among sites. It is therefore important to develop efficient federated GWAS methods that can be used when meta-analysis is not feasible.

5. Sites must put trust in each other to not share the encryption keys with other sites while model parameters are being accumulated.

6. FedGMMAT assumes that the full kinship matrix is available at the Central Server. One can assume that the full kinship matrix is sensitive and it cannot be revealed to the central server as plaintext information. In this case, the sites can resort to assuming that the cross-site entries in the kinship matrix is set to 0. This may be a reasonable assumption in most cases when sites are geologically far apart from each other (e.g., intercontinental collaborations) where the familial relatedness across sites is expected to be low.

References

[1] B. F. Voight and J. K. Pritchard, "Confounding from cryptic relatedness in case-control association studies," *PLoS genetics*, vol. 1, no. 3, p. e32, 2005.
