# Supplementary figures and images for: FedGMMAT: Federated generalized linear mixed model association tests
Source: PLoS Comput Biol. 2024 Jul 24;20(7):e1012142. doi: 10.1371/journal.pcbi.1012142 (PMC11299833; doi:10.1371/journal.pcbi.1012142)

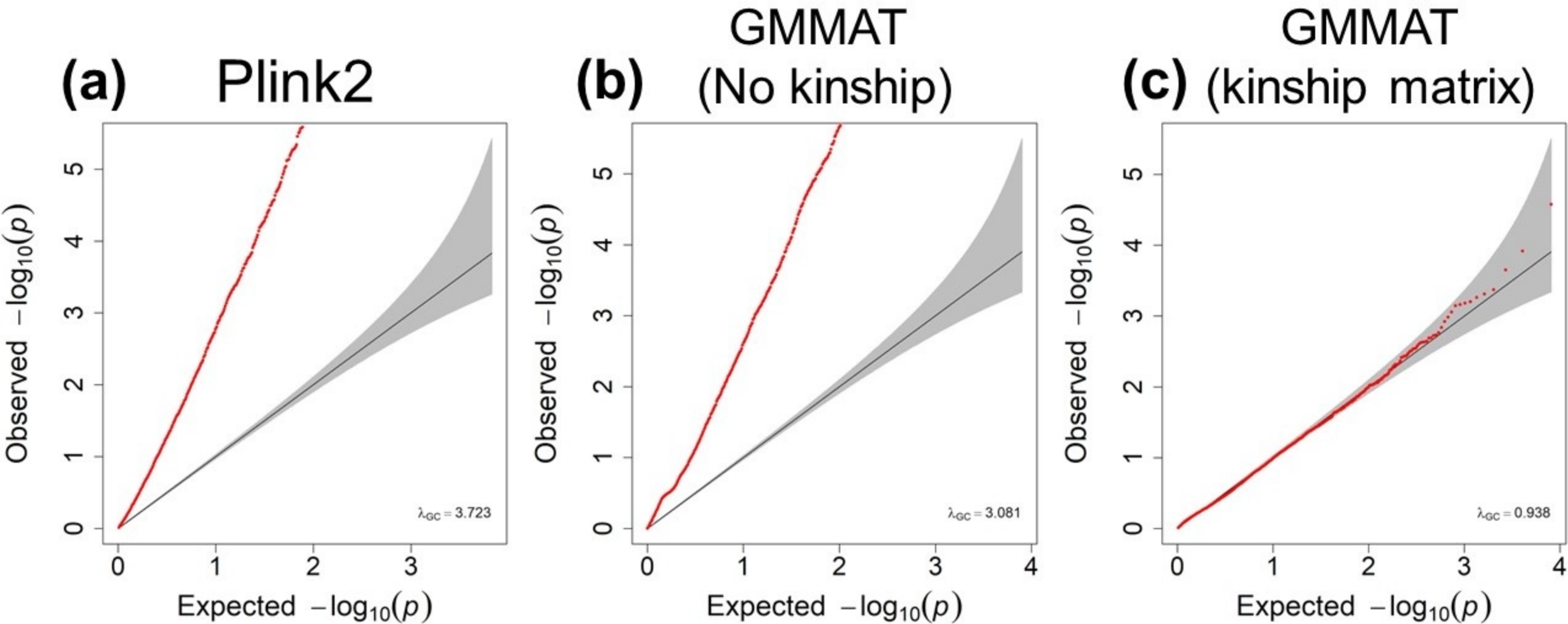

Supplement: S1 Fig — (a) QQ-plot for plink2. (b) QQ-plot for GMMAT with no kinship information (trivial diagonal only kinship matrix). (c) QQ-plot for GMMAT with kinship matrix inferred using SIGFRIED. (PDF) [file pcbi.1012142.s002.pdf]

# Partitioned Noise Aggregation

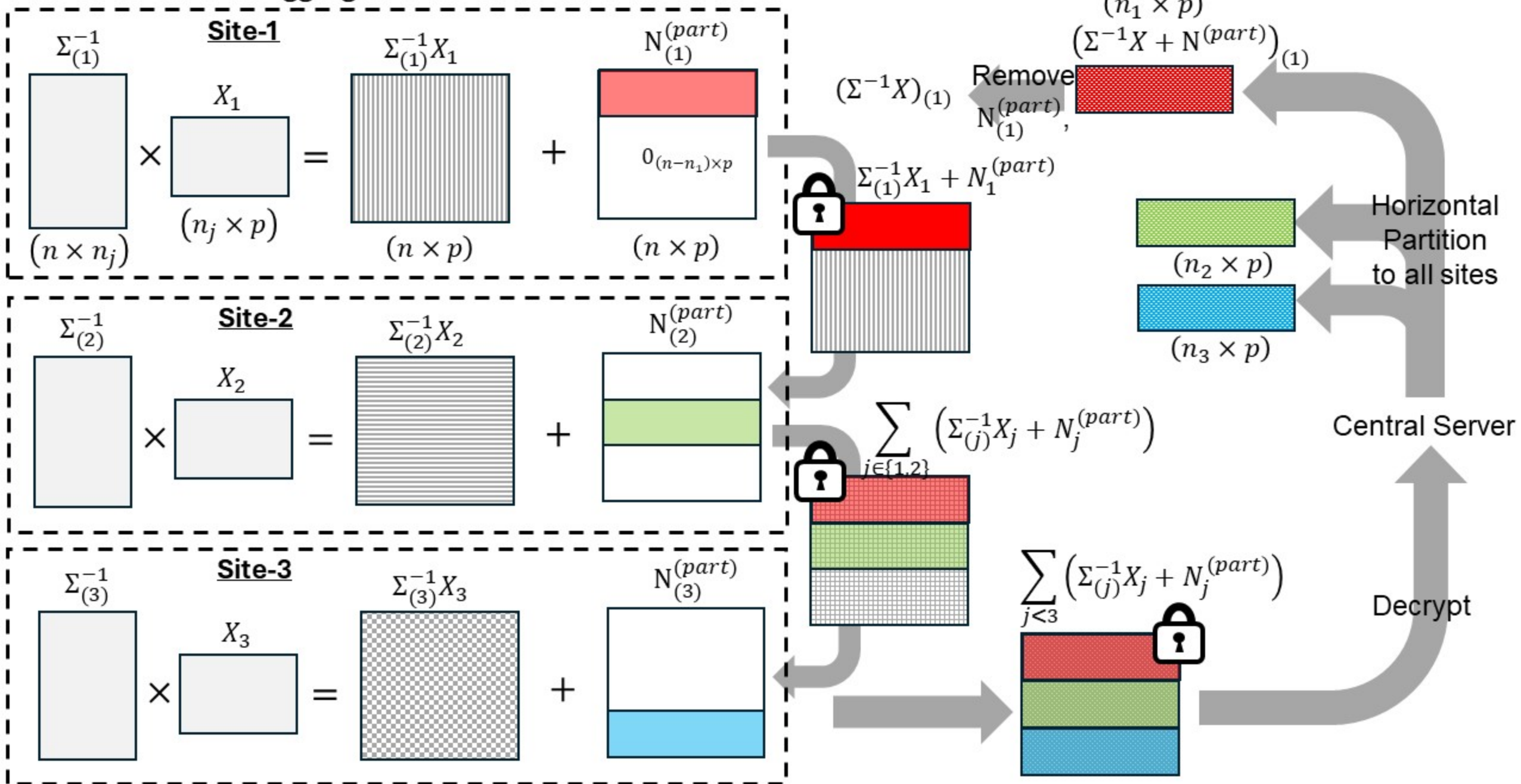

Supplement: S2 Fig — Each site adds partitioned noise matrix to their respective partition. The encrypted matrices are pooled using round-robin schedule and sent the Central Server. Central Server decrypts the data matrix and sends respective partitions to each site. Sites locally remove the partitioned noise matrix from their partition and use the data. (PDF) [file pcbi.1012142.s003.pdf]

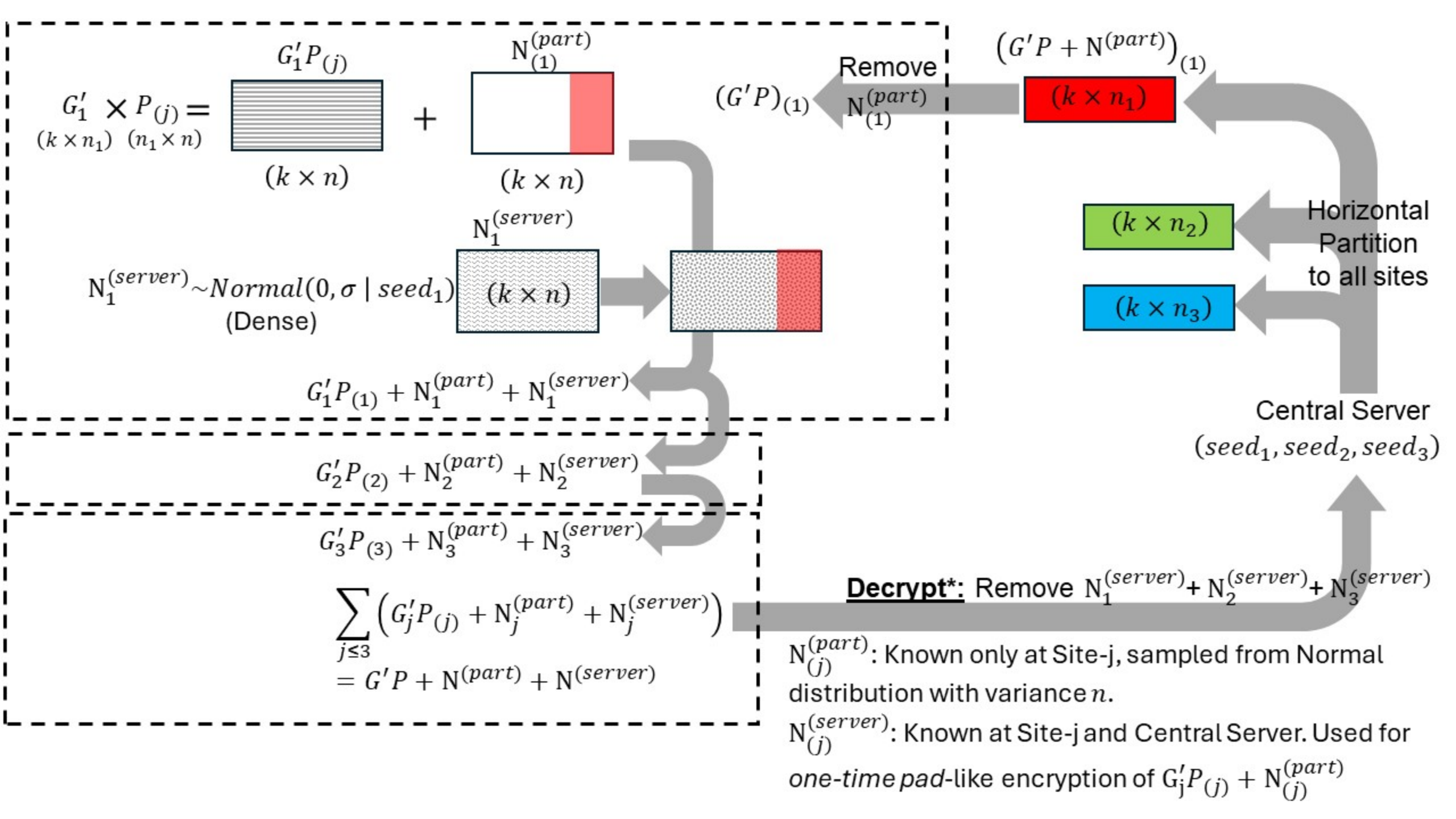

Supplement: S3 Fig — The encryption in each site comprises adding a dense noise matrix that is generated using a secret seed that is shared with the central server. Note that each site adds partitioned noise matrix to their respective partition in addition to the server noise matrices. The pooled noise matrices are removed by the server from the aggregated data matrix. (PDF) [file pcbi.1012142.s004.pdf]
