# Supplementary material for: FedGMMAT: Federated generalized linear mixed model association tests
Source: PLoS Comput Biol. 2024 Jul 24;20(7):e1012142. doi: 10.1371/journal.pcbi.1012142 (PMC11299833; doi:10.1371/journal.pcbi.1012142)
Supplement: S4 Fig — (PDF) [file pcbi.1012142.s005.pdf]

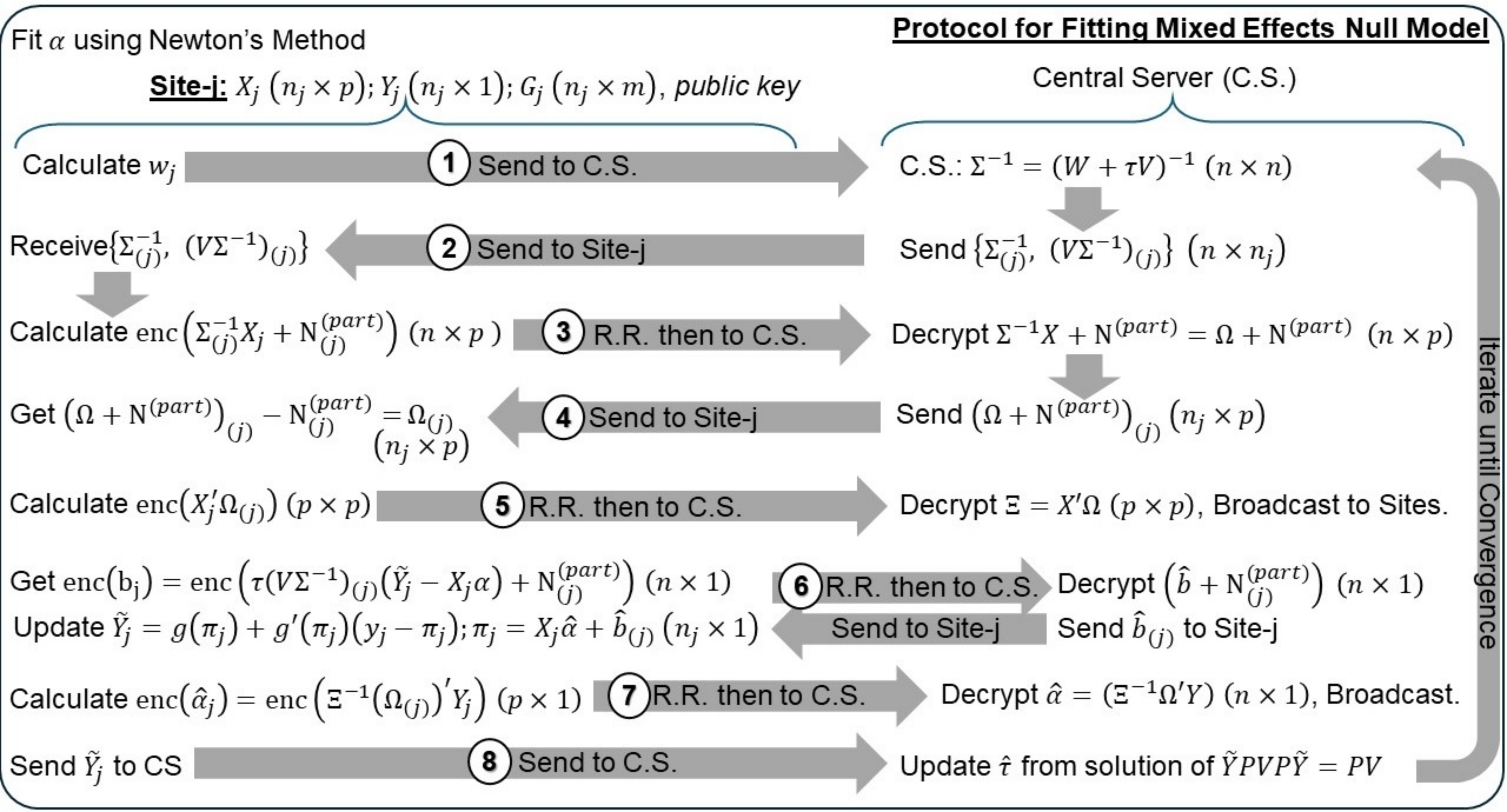

Protocol for Fitting Mixed Effects Null Model
Fit α using Newton's Method
Site-j: $X_j\ (n_j \times p); Y_j\ (n_j \times 1); G_j\ (n_j \times m)$, public key
Central Server (C.S.)
Calculate w_j
1 Send to C.S.
C.S.: $\Sigma^{-1} = (W + \tau V)^{-1}\ (n \times n)$
Receive$\{\Sigma^{-1}_{(j)},\ (V\Sigma^{-1})_{(j)}\}$
2 Send to Site-j
Send $\{\Sigma^{-1}_{(j)},\ (V\Sigma^{-1})_{(j)}\}\ (n \times n_j)$
Calculate $\mathrm{enc}\left(\Sigma^{-1}_{(j)} X_j + \mathrm{N}^{(part)}_{(j)}\right)\ (n \times p)$
3 R.R. then to C.S.
Decrypt $\Sigma^{-1}X + \mathrm{N}^{(part)} = \Omega + \mathrm{N}^{(part)}\ (n \times p)$
Get $\left(\Omega + \mathrm{N}^{(part)}\right)_{(j)} - \mathrm{N}^{(part)}_{(j)} = \Omega_{(j)}\ (n_j \times p)$
4 Send to Site-j
Send $\left(\Omega + \mathrm{N}^{(part)}\right)_{(j)}\ (n_j \times p)$
Calculate $\mathrm{enc}\left(X_j' \Omega_{(j)}\right)\ (p \times p)$
5 R.R. then to C.S.
Decrypt $\Xi = X'\Omega\ (p \times p)$, Broadcast to Sites.
Get $\mathrm{enc}(\mathrm{b_j}) = \mathrm{enc}\left(\tau (V\Sigma^{-1})_{(j)} (\tilde{Y}_j - X_j \alpha) + \mathrm{N}^{(part)}_{(j)}\right)\ (n \times 1)$
6 R.R. then to C.S.
Decrypt $\left(\hat{b} + \mathrm{N}^{(part)}_{(j)}\right)\ (n \times 1)$
Update $\tilde{Y}_j = g(\pi_j) + g'(\pi_j)(y_j - \pi_j); \pi_j = X_j \hat{\alpha} + \hat{b}_{(j)}\ (n_j \times 1)$
Send to Site-j
Send $\hat{b}_{(j)}$ to Site-j
Calculate $\mathrm{enc}(\hat{\alpha}_j) = \mathrm{enc}\left(\Xi^{-1} (\Omega_{(j)})' Y_j\right)\ (p \times 1)$
7 R.R. then to C.S.
Decrypt $\hat{\alpha} = (\Xi^{-1}\Omega' Y)\ (n \times 1)$, Broadcast.
Send $\tilde{Y}_j$ to CS
8 Send to C.S.
Update $\hat{\tau}$ from solution of $\tilde{Y} P V P \tilde{Y} = PV$
Iterate until Convergence
