# Supplementary material for: FedGMMAT: Federated generalized linear mixed model association tests
Source: PLoS Comput Biol. 2024 Jul 24;20(7):e1012142. doi: 10.1371/journal.pcbi.1012142 (PMC11299833; doi:10.1371/journal.pcbi.1012142)
Supplement: S5 Fig — (PDF) [file pcbi.1012142.s006.pdf]

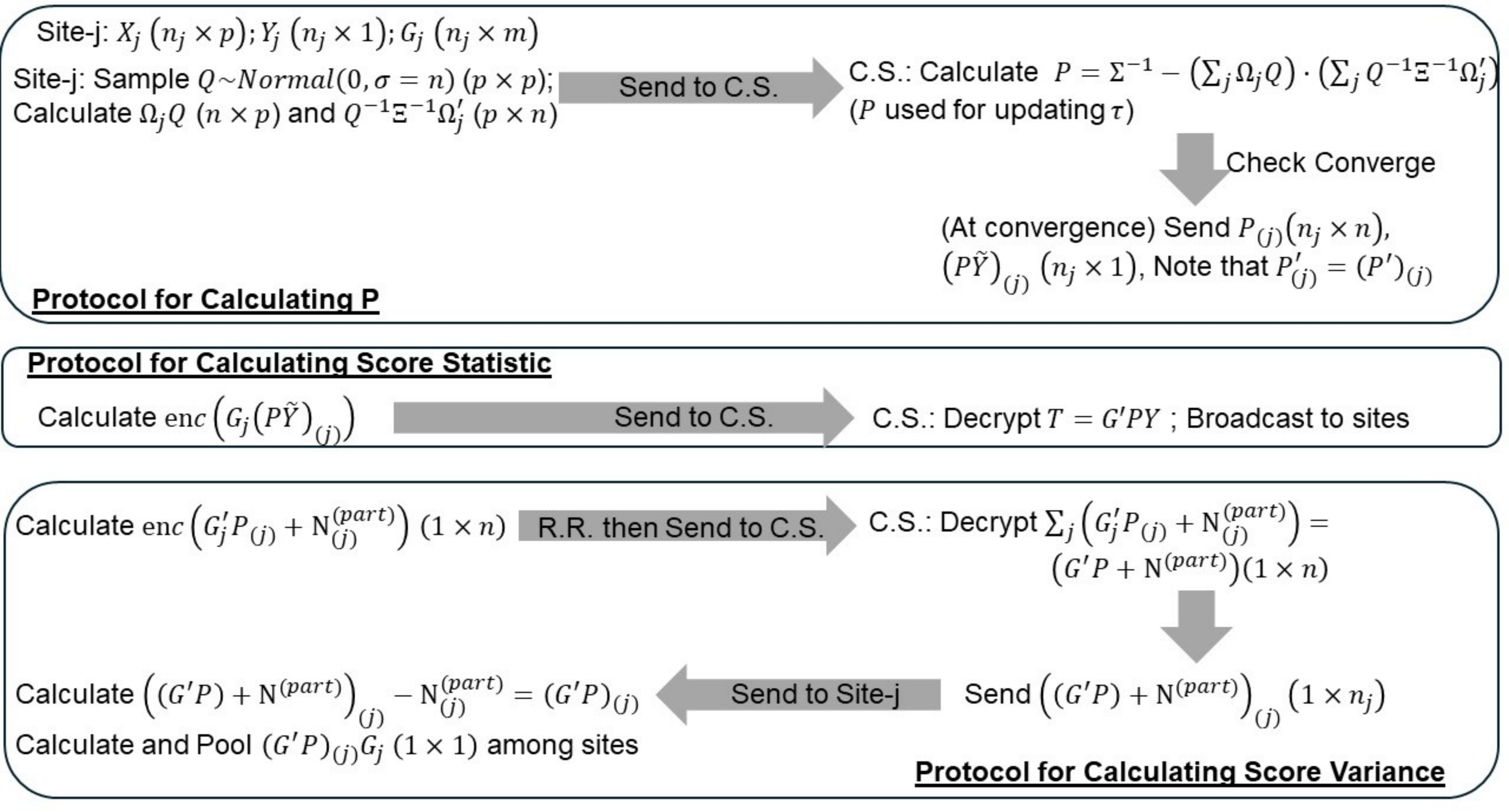
Site-j: $X_j\ (n_j \times p)$; $Y_j\ (n_j \times 1)$; $G_j\ (n_j \times m)$
Site-j: Sample $Q \sim Normal(0, \sigma = n)\ (p \times p)$;
Calculate $\Omega_j Q\ (n \times p)$ and $Q^{-1}\Xi^{-1}\Omega_j'\ (p \times n)$
Send to C.S.
C.S.: Calculate $P = \Sigma^{-1} - \left(\sum_j \Omega_j Q\right) \cdot \left(\sum_j Q^{-1}\Xi^{-1}\Omega_j'\right)$
(P used for updating τ)
Check Converge
(At convergence) Send $P_{(j)}(n_j \times n)$,
$\left(P\tilde{Y}\right)_{(j)}\ (n_j \times 1)$, Note that $P_{(j)}' = (P')_{(j)}$
Protocol for Calculating P
Protocol for Calculating Score Statistic
Calculate $\mathrm{enc}\left(G_j\left(P\tilde{Y}\right)_{(j)}\right)$
Send to C.S.
C.S.: Decrypt $T = G'PY$; Broadcast to sites
Calculate $\mathrm{enc}\left(G_j' P_{(j)} + \mathrm{N}_{(j)}^{(part)}\right)\ (1 \times n)$
R.R. then Send to C.S.
C.S.: Decrypt $\sum_j \left(G_j' P_{(j)} + \mathrm{N}_{(j)}^{(part)}\right) =$
$\left(G'P + \mathrm{N}^{(part)}\right)(1 \times n)$
Send $\left((G'P) + \mathrm{N}^{(part)}\right)_{(j)}\ (1 \times n_j)$
Send to Site-j
Calculate $\left((G'P) + \mathrm{N}^{(part)}\right)_{(j)} - \mathrm{N}_{(j)}^{(part)} = (G'P)_{(j)}$
Calculate and Pool $(G'P)_{(j)} G_j\ (1 \times 1)$ among sites
Protocol for Calculating Score Variance
