# Supplementary material for: FedGMMAT: Federated generalized linear mixed model association tests
Source: PLoS Comput Biol. 2024 Jul 24;20(7):e1012142. doi: 10.1371/journal.pcbi.1012142 (PMC11299833; doi:10.1371/journal.pcbi.1012142)
Supplement: Attachment — Submitted filename: FedGMMAT_PointbyPoint_Response_To_Reviewers.pdf [file pcbi.1012142.s007.pdf]

Dear editor-in-chief, editorial staff, and reviewers,

We would like to thank you sincerely for your time and effort in reviewing our manuscript "*FedGMMAT: Federated Generalized Linear Mixed Model Association Tests*" and for the constructive critiques of the original submission and added new clarifications to the manuscript that have been missed in our original submission. We especially thank the reviewers for their patience in assessing our manuscript and taking the time to point out these critical issues in our presentation and technical content and we are grateful that reviewers have given us a chance to respond to the concerns. In this revision, we have made every effort to address these in the revised manuscript, and we believe this has resulted in an improved presentation. We provide below a point-by-point response to these critiques, with reviewer comments in *italics*, and our response in normal font. Also, for the sake of completeness, we responded to every entry in the decision letter, which may include redundancies. We noted these as best as we can.

- Reviewer #1

Summary:
In this manuscript, the author proposes a federated genetic association testing tool. As the genetic data can never be shared among collaborating sites due to the patient privacy protection regulation, the proposed method also is proposed to protect the intermediate statistics by homomorphic encryption. Through three experiments utilizing both simulated and real-world datasets, the paper demonstrates the method's performance in comparison to the pooled analysis technique. The manuscript is notably well-structured and written in a clear manner. However, it is important to address several existing weaknesses within the content to enhance its overall quality.

Major comments:
1. For experiment 2, it would be helpful if the authors could explain the definition of homogeneous and heterogeneous. What kind of heterogeneity does the proposed method handle and how?
Authors' Response: Thank you for the comment. The homogeneous and heterogeneous dataset denote different simulated datasets used in comparisons in a controlled setting. For the homogenous dataset, the founder subjects are simulated from the Great British (GBR) population of the 1000 Genomes Project. The heterogeneous dataset has multiple populations, namely Great Britain, Mexicans in Los Angeles (MXL), and Yoruban from Ibadan, Nigeria (YRI). We simulated 400 pedigrees, each comprising 16 members using the above populations as the respective founder cohort. From 400 16-member pedigrees, we simulated 6,400 individuals and sub-selected 6,000 individuals divided into 3 sites. We include the comparison of the model performance based on the two different settings to test the robustness of our proposed method.
Effects of Kinship Heterogeneity: In the context of heterogeneity, we clarified the kinship heterogeneity that is handled by the mixed effects models. For this, we performed a new experiment where case and control cohorts comprise outbred and inbred populations. In this

experiment, the genetic contribution to phenotypic variance is assumed to be 0. When we ran the experiments using plink2 (a method that does not handle kinship), we observed that there is very strong bias due to high inbreeding in the case cohort (Fig S1a). Similarly, when we ran GMMAT without kinship information (using a diagonal kinship matrix), we observed the same bias (Fig S1b). When we ran GMMAT with the actual kinship matrix of the cohorts, we observed that the bias was largely corrected by GMMAT (Fig S1c).

Updates to the Manuscript: The descriptions of the homogeneous and heterogeneous datasets and the details of the simulation procedure, including descendant genotype simulation and phenotype simulations, are clarified in "GENOTYPE AND PHENOTYPE SIMULATIONS" Subsection of the Methods Section. Specifically, we also clarified the above description in the Results Section. We also added the results from the heterogeneous kinship analysis results in Supplementary Material under the first Section "Impact of Kinship Heterogeneity on GWAS Results."

2. Can the proposed method also account for the random slope in the model?
Authors' Response: In the current implementation, FedGMMAT only considers random intercepts. This approach stems from the formulation of the mixed effect model that was initially developed for breeding studies[1] and forms the basis of many methods that are used in the field. The basic idea is to consider the genetic effects as fixed effects, while relatedness effects (e.g., cryptic kinship) are regarded as additive polygenic effects defined by a kinship matrix (or an experimental design matrix). The independent environmental effects are modeled as independent Gaussian variables.
While we acknowledge the potential advantages of including random slopes, e.g., the structured heterogeneity in genetic effect sizes can be explicitly modeled, the federated nature of our model introduces unique optimization hurdles that require an extensive re-formulation of the solutions. We therefore believe this would fit into future studies. Nevertheless, we believe this is an exciting extension of the association testing methods where the genotype effect sizes (slope) can be supplemented with a random effect and modeled as a mixed effect.
Updates to the Manuscript: We added the incorporation of the random slope model as a future extension to the Discussion Section.

3. The author built the federated testing procedure based on GLMM. It would be helpful if they discuss any potential limitations or constraints of this model, especially the rationale of using mixed-effect model. It would be also helpful if they explain the random effects in the data of the three experiments, and the potential causes of the existence of these random effects.
Authors' Response: We list below a general list of limitations and criticisms of FedGMMAT that warrant further research in later studies:

1. **Lack of Random Slopes:** As we discussed above, a random slopes model can help further characterize and control confounders. This must be formulated carefully to ensure that the null model is not overly relaxed, which may affect the model's power to detect associations.
2. **Design Constraints in Federated GWAS:** The sites must have designed the study with the same covariate information and the same study design. One of the criticisms (rather than limitations) of FedGMMAT and other federated algorithms is that most studies designed this way would have an accompanying data sharing and analysis plan and can potentially share data among sites rather than performing a pooled analysis. However, this may cause concerns among participants and decrease sample sizes. The studies that would benefit from using federated algorithms may therefore be limited due to the challenges of finding similar study designs.
3. **Requirements of a Federated PCA:** The sites must execute a federated PCA to obtain the most accurate set of population confounding PC covariates. However, the federated PCA approaches in the literature are highly computationally demanding and may fail to correctly identify confounders when there are related individuals in the cohorts. To get around these issues, the sites can use a projection-based approach that we proposed in a previous study, which would also be effective in removing a large portion of the confounding. This approach is also very computationally efficient (one projection only) and is performed only locally. The limitation of this approach is the reliance on an existing large cohort, we foresee that the existence of very large cohorts such as AllofUS and UKBioBank help for this purpose. Numerous studies have pointed out the advantages of using projection-based approaches for federated studies and meta-analysis to partially mitigate biases. We believe that new reference databases can help build comprehensive projection-based approaches.
4. **Comparison with Meta-Analysis:** The potential of using meta-analysis approaches, which already provide the most strongly associated variants in association tests, is already the dominant mode of analysis in performing multi-site and retrospective analysis. However, these advantages also reflect as potential limitations: Numerous studies demonstrated that meta-analysis may bias results when there is heterogeneity among sites. It is therefore essential to develop efficient federated GWAS methods that can be used when meta-analysis is not feasible.
5. Sites must put trust in each other to not share the encryption keys with other sites while model parameters are being accumulated.
6. FedGMMAT assumes that the full kinship matrix is available at the Central Server. One can assume that the full kinship matrix is sensitive and cannot be revealed to the central server as plaintext information. In this case, the sites can assume that the cross-site entries in the kinship matrix are set to 0. This may be a reasonable assumption in most cases when sites are geologically far apart from each other (e.g., intercontinental collaborations) where the familial relatedness across sites is expected to be low.

<u>Source of Random Effects:</u> The random effects generally stem from the correlations or clustering among the subjects, i.e., the subjects are not independently sampled from the population. Of note, the usage of random effect models is standard to correct for the effects of kinship in GWAS. One obvious reason for the relationships among subjects is the familial relatedness among subjects. Due to the polygenicity of the diseases/biological conditions, we expect that correlated genetic information will lead to a correlation in the phenotypic data, which may bias the calculations in case there are large families with large amount of shared genetic information. In principle, one could remove these families (as plink does) to minimize these biases; this, however, decreases the power of detecting new variants. GMMAT models these effects using a "kinship matrix" as the covariance of the random effect and the effect size is estimated by the algorithm. Note that familial relatedness is not the only source of random effects. Another example is multiple measurements from the same individuals and correlated effects (e.g., longitudinal studies with repeated measurements, and shared environment effects). Note that FedGMMAT can seamlessly accommodate longitudinal experiments (i.e., multiple random effects). However, this requires several modifications to the algorithm that may further increase the resource requirements. We added a new simulation that demonstrates the effect of kinship and relatedness on the design matrices.

<u>Updates to the Manuscript:</u> We have added a new section to the Supplementary Material discussing these limitations to ensure brevity in the main text. We also added a note to the discussion summarizing these limitations.

4. It would be good to see this algorithm to be utilized in a real-world collaborative study scenario. This would be good opportunity to demonstrate the validity and feasibility of the method's utilization in a real multi-site collaboration.

<u>Authors' Response:</u> We agree that a real-world collaborative study would be a great opportunity to demonstrate the validity of our method. This type of demonstration would also exemplify use-case scenarios and would be very helpful for revealing potential hidden challenges, such as quality control issues among sites, but we do not anticipate these cases. Unfortunately, we could not perform a real multi-site collaboration since it was not possible to establish a collaboration in the given time period. We have, however, conducted a large-scale simulated study using 32,000 subjects and 1 million variants among 3 sites. We discuss these results below in response to Reviewer 2's 2nd major comment, and we hope that Reviewer can refer to these results for our complete response. In a nutshell, we showcase that FedGMMAT can be executed with moderate memory and time requirements on this scale of collaboration. We also propose some number of extensions that can improve time and memory usage performance, e.g., cloud-based runs to decrease network latency and cost, using optimized schedules instead of round robin to decrease run times, and we also presented new lightweight *One-Time Pad-like* protection strategies to efficiently protect the data.

Updates to the Manuscript: We have included the large-scale run results under the "Communication cost performance" subsection of the Results Section. We added two tables summarizing the network usage. We also included a new figure showing how the model fitting scales with the number of subjects in the datasets.

5. This is a lossless distributed learning algorithm for score test on genetic data. Li R, Duan R, Zhang X, Lumley T, Pendergrass S, Bauer C, Hakonarson H, Carrell DS, Smoller JW, Wei WQ, Carroll R. Lossless integration of multiple electronic health records for identifying pleiotropy using summary statistics. Nature communications. 2021 Jan 8;12(1):168. What's the difference between your method and theirs?

Authors' Response: We reviewed this reference. Following are the main differences:

1. Li et al. does not focus on performing association testing but more on the pleiotropy analysis from phenome-wide association testing PheWAS results, i.e., single variant effecting multiple phenotypes. In principle, the random effects would similarly impact these methods as well, however, the authors did not include these sources in this study, e.g., there may be relatedness among individuals in different sites.
2. Li et al.'s approach is a meta-analysis approach for detecting pleiotropy. This is inherently a different task from FedGMMAT, which aims to perform GWAS using datasets from multiple sites.

Updates to the manuscript: We included this reference with other methods requested by the first reviewer.

6. How many iterations of updating model parameters (i.e., transferring aggregated information) are required in three experiments? It would be helpful if the author can discuss the feasibility of the implementation of this method in the real-world setting.

Authors' Response: We have now updated the number of iterations that are required to update model parameters in the three experiments in Tables 5-6, and Figure 5. On average, the fixed model fitting requires 10 iterations, and random effect model fitting requires 12 iterations. Overall, we observed that the models converge in a small number of iterations.

We now included a new experiment that simulates 32,000 subjects and 1 million variants and recorded the resource requirements (Tables 5 and 6 are included below). Overall, the OTP-like encryption-based efficient algorithm finishes in 2.5 days, and the HE-based encrypted model completed in approximately 5 days, which indicates moderate-to-high resource requirements. The main chunk of this computational requirement stems from the scoring step, where the variance matrix of the score test is aggregated among sites, i.e., $diag(G'PG)$. In this part of the protocol, the algorithm encrypts ${G'}_j P_j$ which is a $k \times n$ matrix for each of the k variant chunk before it is decrypted at the server. Here P_j $(n_j \times n)$ denotes the horizontal partitioning of the projection matrix that is calculated at the server. We discuss the numerous approaches that we used to decrease the resource requirements while ensuring that data protection is not compromised:

1) **$G'_j P_j$ Aggregates Genotype Information.** One of the main observations is that each site receives only a distinct horizontal portion of the full projection matrix P_j and does not receive other sites' projection matrix shares. Secondly, in $G'_j P_j$ is a $k \times n$ matrix, however, it does not immediately leak individual level genotype information since site j's P_j ($n_j \times n$) mixes the genotype among n_j subjects and aggregates among n subjects. Thus, the genotype information is inherently aggregated in $G'_j P_j$ components. This matrix can, therefore be deemed to be safe to share without encryption among sites. This decreases resource requirements by 10-16 fold at each chunk (Table 5). We now showed that this usage can be further decreased by quantizing these matrices.
2) **One-Time Pad (OTP) like encryption and partitioned noise matrices help efficiently protect variant allele frequency leakage from $G'_j P_j$.** It is, however not immediately possible to share these matrices since the rows of $G'_j P_j$ leaks the variant level allele frequencies and may reveal rare variant information. To protect this information, we can use HE for protection, however, using HE is too demanding for this step since it increases computational and storage requirements extensively. We now present an alternative to HE in the scoring step using the One-Time Pad like encryption: Central server shares a noise matrix of size ($k \times n'$) that can be added to $G'_j P_j$ matrix by the sites (or a randomly selected site at each iteration). After $G_j'P_j + noise_{k\times n}$ are aggregated among sites, the central server can remove the aggregated $noise_{k\times n}$ and send aggregated $G'P$ to all sites for further processing after partitioning it (More clear explanations in Fig. S2, S3 and protocol steps in Figs. S4, S5). Note that these site-specific noise matrices are like symmetric encryption keys and are known only to central server and to the individual sites using a pseudorandom number exchange among sites and central server (i.e., one scalar value). We have now clarified the secure aggregation protocol description, and included usage of the site-wise partitioned noise matrix and the OTP-like encryption approach to protect the matrices. These protocols are used in aggregation operations and help protect data leakage to the central server (via partitioned noise matrix) and individual sites (via homomorphic encryption or one-time-pad encryption between individual sites and central server). These approaches enable flexibility in terms of efficiency/privacy tradeoffs and help build new algorithms. We have now used One-Time Pad like encryption (Fig. S2, S3) as an alternative to Homomorphic encryption for protecting the data).
3) **Permutation Protects Variants at Central Server.** The sites can choose to include a permutation operation over all variants (i.e., $TG'PGT'$) to ensure that the variant ordering is not revealed to the server. This is a fairly straightforward operation and can be accomplished by exchanging pseudo-random shares and shuffling variants at each site before $G'P$ matrices are sent to the central site. This is, however, not necessary when sites make use of the partitioned noise matrices.

Updates to the Manuscript: We added the OTP-like encryption strategy to the Methods Section as an alternative to HE-based encryption for variant scoring. We also updated the Methods section

to include protection of the site's partitioned matrices in the aggregation mechanisms using the partitioned noise matrices. Supplementary Information is updated to include a detailed description of the steps of the protocols used in model fitting and scoring steps with 5 new figures.

Minor comments:

1. On Page 3, I assume that HE means homomorphic encryption. However, this is not defined in the previous section or in the figure legend.

Response: We updated the abbreviation of HE to "Homomorphic Encryption".

2. On Page 5, section B, the Appendix equation is not correctly referred.

Response: We updated the equation reference number.

3. On Page 7, section E Step 5, the equation is not correctly referred. Please check through the manuscript. There are other places with the same issue.

Response: We have reviewed the whole manuscript to ensure the equation numbers are correctly referred.

- **Review #2**

Summary:

This paper presents FedGMMAT, a federated algorithm designed for generalized mixed model association tests. FedGMMAT extends the GMMAT algorithm by adapting it for federated analysis. This involves reconfiguring the algorithm to compute specific intermediate terms, including gradients, Hessian, and site-specific parameters, locally at each site. These terms are then securely aggregated across sites using homomorphic encryption. To facilitate this process, a trusted computing entity decrypts the aggregated statistics to perform global updates to the model. Additionally, FedGMMAT employs a two-step procedure for association testing. Initially, it trains a null model that includes only covariates. Subsequently, it calculates association p-values using score tests based on the null model. The effectiveness of the proposed algorithm is evaluated using three datasets: a small synthetic dataset (400 samples), the 1000 Genomes dataset with simulated phenotypes (6000 samples), and a real genotype-phenotype dataset from dbGaP (2545 samples). The results indicate that FedGMMAT produces comparable results to those obtained with GMMAT. Although this work offers technical insights into federated GLMMs, its practical applicability remains uncertain due to privacy protection and computational cost concerns.

Major comments:

1. The practical viability of the proposed method in its intended settings is uncertain due to privacy issues associated with the exposure of intermediate statistics through FedGMMAT. Federating the analysis does not inherently ensure data protection. This manuscript makes exaggerated claims that may be misleading to readers. For instance, statements such as "[FL] complies with the regulations on PHI protection" and "intermediate statistics are protected by homomorphic encryption" are presented without adequate support. The manuscript lacks a thorough discussion

of privacy protection provided by the proposed method or justification for the intermediate statistics that are disclosed, which is crucial for regulatory compliance and data protection:

Authors' Response: We acknowledge that the reviewer's concerns are valid and we have unintentionally overstated certain parts of the introductory portions of the manuscript. We reviewed these sections and made sure they are well justified with references to the literature. We also removed the overstated sentence "*[FL] complies with the regulations on PHI protection*". We include more discussion in the following comments below for the updates we include. We also included a more extensive discussion about the privacy considerations and limitations of FedGMMAT. Overall, we use homomorphic encryption and combine it with site-specific noise addition to protect individual-level information between sites and also from the central server. We do acknowledge that the central server gains access to a proxy for the binary phenotypes (without other identifiers), the kinship matrix, and the null model parameters.

Central Server as an Entity: Central Server is assumed to be a trusted entity that executes the protocols as agreed upon (e.g.,[2]), without the necessity of storing identifying information from the sites (i.e., does not ned to store registration and identity information). Since Central Server is a trusted authority, the main concern regarding Central Server is the event of hacking or accidental leakage of the datasets from the server. We discuss these cases below.

Kinship Matrix: Revealing the kinship matrix to the Central Server may or may not create privacy concerns for the sites as described in their data usage agreements. In essence, a kinship matrix is a proxy for measuring relatedness between individuals. While it provides some indication of familial pedigrees, it cannot immediately be used to build familial lineages without other information (i.e., an individual's half-sibling and aunt have the same kinship coefficient with the individual, similarly, an individual's niece (or nephew) and uncle have same kinship coefficient with the individual. Thus, it is not reliable for uncovering family pedigrees to match existing pedigrees. The kinship matrix depends on the subjects in the cohort and is not a fixed re-identifying information, i.e., the rows (or columns) of the kinship matrix is not a unique signature for each individual that is invariant between different kinship matrices that harbor the same individual. Also, most kinship matrices are extremely sparse with the exception of the familial studies, which include a uniform selection of families (e.g., trios). Thus, we assume that the kinship matrices are not of immediate concern even if they are leaked unless other re-identifying information is leaked with them. One exception is datasets with families comprising large numbers of children, which may by themselves be re-identifieable due to being outliers. These extremely outlier sized families can be: 1) kept out of the studies or 2) can be considered with families of similar sizes only or 3) can be subsampled and used in a proxy GWAS setting.

Revealing of $\widetilde{Y}$ (working phenotype): $\widetilde{Y}$ is sent to the Central Server while the variance component is being updated and at the beginning of the score statistic assignment at the second stage of FedGMMAT. The main concern around leaking of $\widetilde{Y}$ is combining it with kinship matrix. Although a curious entity may be able to build partial family trees and assign phenotypes, it is not immediately clear how these can be used for re-identifying individuals since most families will be of similar size with no immediate way to distinguish them in terms of phenotypic patterns.

Furthermore, the entities do not know the phenotype that is being studied (this information does not have to be revealed). Finally, $\widetilde{\boldsymbol{Y}}$ is a vector that is used in two places in FedGMMAT and it can be re-submitted and discarded after they are used.

$\widetilde{\boldsymbol{Y}}$ can be protected using the protected aggregation mechanisms: Of note, our current mechanisms can be used to protect $\widetilde{\boldsymbol{Y}}$: To update tau, the central server calculates $\widetilde{\boldsymbol{Y}}'\boldsymbol{Z}\widetilde{\boldsymbol{Y}} - \boldsymbol{PV}$ where $\boldsymbol{Z} = \boldsymbol{PVP}$ (Protocol for Fitting Mixed Effects Null Model in Figure S4). Notice that calculation of $\widetilde{\boldsymbol{Y}}'\boldsymbol{Z}\widetilde{\boldsymbol{Y}}$ is very similar to $\boldsymbol{G}'\boldsymbol{PG}$ (Protocol for Calculating Score Variance in Figure S5) and can be calculated using a similar protocol. This mechanism requires exchanging the horizontal partitions of $(\boldsymbol{PVP})_{(j)}$ matrix among sites. Secondly, $\widetilde{\boldsymbol{Y}}$ is used in calculating score statistics for all SNPs (Protocol for Calculating Score Statistic in Figure S5) as $T = G'P\widetilde{\boldsymbol{Y}}$. This quantity can be calculated by first pooling $\boldsymbol{G}_{\boldsymbol{j}}'\boldsymbol{P}_{(j)}$, and then pooling $(\boldsymbol{G}'\boldsymbol{P})_{(j)}\widetilde{\boldsymbol{Y}}_{\boldsymbol{j}}$. where $(\boldsymbol{G}'\boldsymbol{P})_{(j)}$ is the vertical partitioning of $\boldsymbol{G}'\boldsymbol{P}$ matrix.

While we provide certain measures to protect above risks, we do acknowledge these limitations of FedGMMAT.

Updates to the Manuscript: We have included the discussion points above in a new subsection named “Privacy and Security of Federated Protocol”.

- There seems to be an exposure of individuals' phenotype residuals ($\tilde{y}$) to the trusted computing entity through FedGMMAT. Given that many phenotypes are considered PHI, it raises the question of whether such a practice is feasible.

Authors’ Response: As we discussed above, our updated set of aggregation mechanisms can be used to protect $\widetilde{\boldsymbol{Y}}$ information, albeit with an increased cost of communication between parties. From this perspective, the studies that require protection of $\widetilde{\boldsymbol{Y}}$ can utilize protected aggregation mechanisms to protect $\widetilde{\boldsymbol{Y}}$.

We also would like to point out that $\widetilde{\boldsymbol{Y}}$ is a proxy for the real phenotype, however it is not immediately possible to link $\widetilde{\boldsymbol{Y}}$ to an individual since $\widetilde{\boldsymbol{Y}}$ is one scalar value for each individual and cannot be immediately used for re-identifying an individual, i.e., there are possibly thousands of cases and controls in the datasets which do not immediately reveal an individual’s identity. Even with the kinship matrix, which reveals size and number of families in the study, there are possibly hundreds of families with similar sizes and relatedness patterns (e.g., trios, siblings etc). Thus, it does not immediately leak any individual-specific information. We therefore do not encrypt the phenotype residual ($\widetilde{\boldsymbol{Y}}$) since we want to increase the computational efficiency and the phenotype residual does not leak much information.

Updates to the Manuscript: We have summarized the above points to the new subsection named “Privacy and Security of Federated Protocol”.

- Iterative updates to models, such as gradients and Hessian computations, may pose a risk of data leakage. This potential concern should be explored more comprehensively, and if possible, mitigations should be introduced and discussed.

Authors' Response: Hessian and Gradients are encrypted via Homomorphic encryption in the secure aggregation step. While these model parameters and updates are broadcast in plaintext format, this information is of miniscule size (*p* size vector of gradients and *pxp* matrix of Hessian) since the null model fitting step consists only of covariate model fitting. For example, given 20 covariates, only 420 values (20 Gradients+400 Hessian) are revealed to the central server and 20 plaintext values are broadcast to all sites at each iteration whereby the model converges quickly in consecutive iterations. Given that these updates are calculated from thousands of samples, we believe it is highly improbable that a potential leakage can reliably and consistently identify the participation of outlier samples. We nevertheless added this as a potential route to leakage in the discussion.

Updates to the Manuscript: Above points are added to the Subsection titled "Privacy and Security of Federated Protocol".

- While the manuscript mentions the possibility of using other secure kinship estimation methods to compute the genetic kinship matrix (V) across sites, it does not address the feasibility of releasing kinship information for pairs of individuals between sites. It needs clarification whether FedGMMAT can still be applied when V cannot be disclosed.

Authors' Response: In the current version, only the Central Server has access to the complete kinship matrix. It is assumed that the sites only know the corresponding rows/columns of the kinship matrix, i.e., site-j has calculated the corresponding j'th horizontal and vertical partitions of the kinship matrix. To clarify this potential limitation, we have now acknowledged this as a limitation of FedGMMAT. In case when the kinship matrices are not shareable among sites, FedGMMAT can be applied using site-specific kinship matrices and excluding cross-site familial relationships, i.e., assuming that there are no relatives across different sites by setting the corresponding kinship entries to 0. In most cases, this can be a reasonable assumption since kinship between different sites will be negligible apart from the low amount of cryptic relatedness. While genetic kinship could be construed as sensitive information, the kinship matrices generally do not reveal exact familial pedigree information. New methods can also be used to securely calculate kinship information among sites[3] although they may need to be tuned for populations of interest. We are currently working on extending the scale of our approaches for larger datasets.

We also would like to highlight that the separation of kinship estimation and association testing is important when admixed populations are analyzed. In these cases, genetic relatedness matrices may fail to dissect the familial relationships from population stratification effects[4]. However, there is no clear consensus on how to execute GWAS in admixed populations in the field yet. We therefore believe alternative approaches can help increase design choices and increase usability in the field.

2. The manuscript falls short in providing a comprehensive evaluation of the algorithm's runtime performance. Section II-D, which addresses communication cost performance, merely mentions that the size of the SNPs subset can impact computation time without discussing the applicability of the proposed method to large GWAS datasets or how the runtime scales with the number of samples. A particular concern arises from the proposed method's direct use of the genetic kinship matrix, which may result in limited scaling behavior.

Author's Response: We have now added a new experiment where we simulated 32,000 subjects with 1 million SNPs as a more realistic test case scenario. For simulating the subjects, we used 2,000 16-member pedigrees of 3 generations with 8 founders and 8 descendants[5]. The simulations were performed using three European founder populations (GBR, TSI, FIN). We used 11 covariates (1 random assigned gender and 10 population PCs) and the binary phenotype is simulated using 5000 randomly selected causal variants with effect sizes sampled from $N(0, 0.5)$ (Methods Section). Random environmental noise standard deviation was set to 0.5. We divided the dataset among 3 sites and executed FedGMMAT on the same computer (96 core Intel Xeon Platinum 8168 CPU clocked at 2.7GHz) such that each site is run in order. We believe this is a fair test since the computation heavy protocol comprises round-robin-based updates to parameters. We assume that the sites use an AWS-based cloud system where shared volumes are mounted on instances that are running FedGMMAT among sites for efficient sharing of intermediate datasets. This can be accomplished using Amazon Web Services' *EBS multi-attach*, which is a very efficient way of sharing data in the cloud environment.

Regarding Scaling with Kinship Matrix Usage: We agree with the reviewer's concern that kinship matrix estimation is computationally demanding. It is, however, worth noting that there are new approaches that use efficient protection mechanisms (e.g., orthogonal projections and using multiplicative noise to protect genotype matrices) to perform large scale kinship estimation at 10,000 subject scale. In our experience, the pairwise kinship estimation can be potentially outsourced to a trusted kinship estimation server (potentially a separate service) where sites submit an anonymized genotype matrix that is corrected for kinship estimation (needed for kinship estimation) wherein the variant coordinates are shuffled and individual identifying identifiers are removed and matrix is rotated by an orthogonal matrix on the right and left by the collaborating sites. This ensures that actual variant positions are not known and that the genotype information is obfuscated. This server would simply calculate the inner product of the rows and columns of this matrix. Notice that kinship matrices are extremely sparse matrices, which is generated by thresholding the dense matrix with a cutoff (https://saigegit.github.io//SAIGE-doc/docs/createSparseGRM.html). Even for UKBiobank, the kinship matrix is more than 99% sparse and can be shared around sparsely. Thus, the matrix can be stored in sparse matrix format and be processed using sparse matrix libraries that offer high performance improvements.

Other Relatedness Matrices can be accommodated by FedGMMAT: We would like to also highlight that, other non-genetic relatedness matrices (e.g., environmental factors or longitudinal experimental designs) are also very sparse and can be used in FedGMMAT's setup in the future.

This is an interesting unique advantage of using an independent relatedness matrix to model the random effects in GWAS testing.

Large Scale Run Results: We tested two versions of the algorithm; one that uses homomorphic encryption (HE) in each step and one without HE (non-HE) for each step (Note that the non-HE version represents the resource requirement for the new *One-Time Pad*-like, OTP-like, lightweight protection mechanism). Overall, non-HE algorithm completed in *2 days, 16 hours, 18 minutes* and HE version completed in *5 days, 10 hours, 31 minutes*. To evaluate the steps take the most amount of time in HE-based calculations, we benchmarked the scaling of run-time in null model fitting step. We observed even with HE, the model fitting takes around 850 seconds, i.e., 15 minutes (Fig. 5), which indicates that virtually the whole algorithm is spent in calculating the score test for the 1 million variants. Given that there is a large difference between HE and non-HE versions, we reasoned that more efficient security measures can benefit efficiency. An important observation is that we use FHE library only for addition operations in aggregations, which incurs high computational cost.

OTP-like Encryption Mechanism. To mitigate this cost, we updated the aggregation operations to use site-specific partitioned noise and replaced HE with a *One-Time Pad*-like (OTP-like) encryption of data using server-generated noise (for each site) such that it can be aggregated after adding noise without revealing it to the server or the other clients (Fig. S2, S3). In essence, FedGMMAT makes extensive use of aggregate-then-partition mechanism in calculations, which require simple matrix summations. To "encrypt" the full matrix, we present usage of site-specific full dense noise matrices (i.e., similar to One-Time Pads), which are known to each individual site (and different for each site) and the central server. This is followed by the addition of the site-specific partitioned noise matrix, which is known only by each site. After aggregation, the server removes the site-specific noise matrices (i.e., "decrypts" the OTP-like encryption), partitions the aggregated matrix, sends the partitions to sites (Fig. S3). Finally, each site removes its own share of partitioned noise matrix locally to obtain the site-specific aggregated partition. Overall, each aggregation operation is protected by a full noise matrix of OTP-like encryption (known by each site and the central server); and the partitioned noise matrix known only by each site. This protocol has the same trust requirements among the sites wherein the central server is assumed to be trusted by the sites to not deviate from the protocol.

This modified approach is advantageous since computational complexity is equal to the plaintext (non-HE) version, except for the 2 extra matrix addition operations to add noise matrices at each site. Furthermore, the network requirements of this approach do not entail ciphertext expansion (approximately 16 times increase in comparison to the original matrix sizes) that is observed in HE-version (to be described below). We therefore expect that the OTP-like method can substantially increase performance for large scale calculations with FedGMMAT as an efficient alternative to HE at the scoring step.

We now updated the presentation of variant scoring step to include the new protocol details in the main text. We present the non-HE as a proxy for the OTP-like encryption strategy, which we believe is a fair representation of the algorithm's performance.

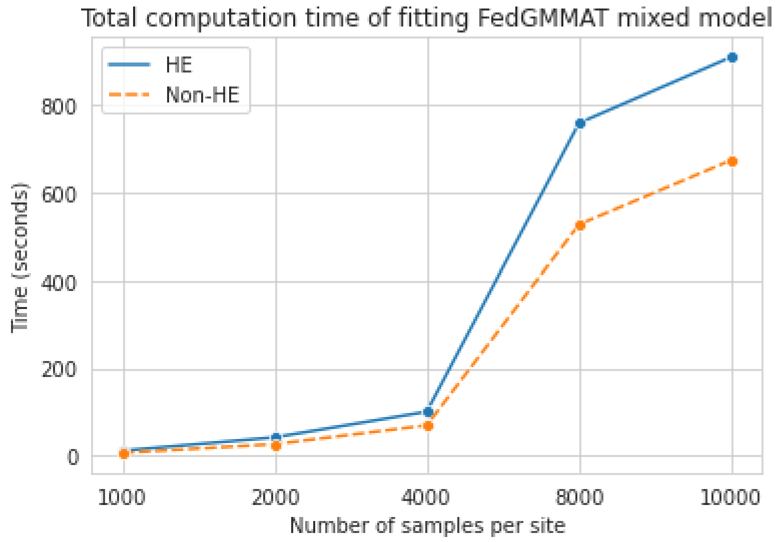

Network Usage: We next studied the usage of storage resources for network transfers, which makes up an important part of performance. We divided the total data transfer size for each step of the model fitting and score testing steps for HE and non-HE versions (Tables 5,6). Non-HE version is included as a proxy for the OTP-like encryption version.

Network Usage for Null Model Fitting: In total, the server-client uses 61 gigabytes of data transfer for fitting the null model with 12 iterations of the model fitting stages. The encrypted data from clients to the central server takes up very small network bandwidth at this stage (Tables 5,6). The most bandwidth is spent exchanging $V\Sigma^{-1}$ and Σ^{-1} matrix partitions from central server to the sites.

Network Usage for Scoring Test: HE and non-HE (i.e., OTP-like) versions require approximately 3.66 terabytes and 359.2 gigabytes, consecutively. The highest network transfer is used by G'P matrix passed from clients to the server. Of note, the OTP-like encryption strategy enables a substantial decrease in the network requirements compared to HE in scoring stage.

Quantization of Large Matrices: One approach to improve performance (memory usage, network usage) is to quantize large matrices. For example, in the scoring stage, we can quantize the $\left(G'P + N^{(part)} + N^{(server)}\right)$ matrix from 64-bit precision down to 32-bit precision before it is sent for aggregation when OTP-like protection scheme is used. This approach effectively halves the storage and network requirements. To test quantization of $(G'P)$ matrix in scoring, we quantized G'P matrices to 32-bits and ran scoring of randomly selected 200-variant chunk. The final p-values of the variants matched with more than 0.999 Pearson R2 correlation to the original results. This result indicates that the scoring stages can be run with quantized matrices using OTP-like protection of the matrices. At the null model fitting, similar quantization can be applied to the

scoring stage where partitions of Σ^{-1} and $V\Sigma^{-1}$ are sent over to the clients, which take up the most amount of space. We also tested quantizing these matrices to 32-bit representations (i.e. `float32` type) in model fitting steps and observed that there is again virtually no loss of accuracy in the predicted null model parameters compared to 64-bit representations.

Usage of Cloud Resources can improve performance with little cost overhead. We recommend using cloud-based storage among collaborating institutions where sites can cross-mount shared volumes (Using, for example, Amazon Web Services' *EBS multi-attach*) for sharing of the large intermediate datasets.

If cloud usage is not viable, our results indicate that the HE-based null model fitting followed by the scoring strategy that uses OTP-like protection with 32-bit quantized matrices can be accomplished in around 210 Gbytes of data transfers per client (30.5 Gbytes for model fitting and 179 Gbytes for scoring test), which we believe can be practically run over high speed networks among institutions using a shared disk space (e.g., COLLAGENE's file transfer utilities) or via direct communications.

The table (Table V in main text) below shows the network traffic from each client to the server. This information is encrypted by OTP-like encryption or HE encryption and is protected by the site-specific partitioned noise matrices.

	Client to server	Data size	Non HE (OTP-like) encryption memory size (MB) per client	HE memory size (MB) per client
Null model	$(\sum_{j=1}^{J} \tilde{\ell}_j^{(t)})$	p-dim vector	4.577E-5	0.0344
	$(\sum_{j=1}^{J} \mathbf{H}_j^{(t)})$	$p \times p$ matrix	0.0005	0.4124
Mixed model	$(\sum_{j=1}^{J} \boldsymbol{\Xi}_j)$	$p \times p$ matrix	0.0010	0.4125
	$(\sum_{j=1}^{J} \boldsymbol{\Omega}_j)$	$n \times p$ matrix	2.9297	38.7689
	$(\hat{\boldsymbol{b}}_j)$	n-dim vector	0.2441	3.2999
Score test	$(\mathbf{G}_j^{\top}(\mathbf{P}\tilde{\mathbf{Y}})_{(j)})$	$nSNPs$-dim vector	7.6294	103.1208

	$G_j^\top P_j^\top$	$k \times n$ matrix	48.828125 (k=200)	644.6279 (k=200)
	GP_jG_j	$k \times k$ matrix	0.3052 (k=200)	4.1245 (k=200)

Following table summarizes the network traffic from Server directly to the clients and are not encrypted:

	Server to clients	Data size	Non HE (OTP-like) encryption
Null model	$\boldsymbol{\alpha}^{(t)}$	p-dim vector	9.1553E-5
Mixed model	$(\mathbf{V\Sigma}^{-1})_{(j)}$	$n \times n_j$ matrix	2604.1667
	Σ^{-1}	$n \times n_j$ matrix	2604.1667
	$\boldsymbol{b}^{(t)}$	n-dim vector	0.2441
	$\hat{\tau}$	scalar	3.8147E-6
Score test	$(\mathbf{P\tilde{Y}})_{(j)}$	n_j-dim vector	0.0817
	P_j	$n \times n_j$ matrix	2604.1667±11.9809
	GP_j	$k \times n_j$ matrix	16.2760±4.1245 (k=200)

Encryption Strategy	Spearman correlation	Pearson correlation (R2)
Non-HE (OTP-like encryption for scoring)	1.0	0.9991
HE	1.0	0.9787

Correlation between the P-values assigned by GMMAT and FedGMMAT for 1 million SNPs dataset.

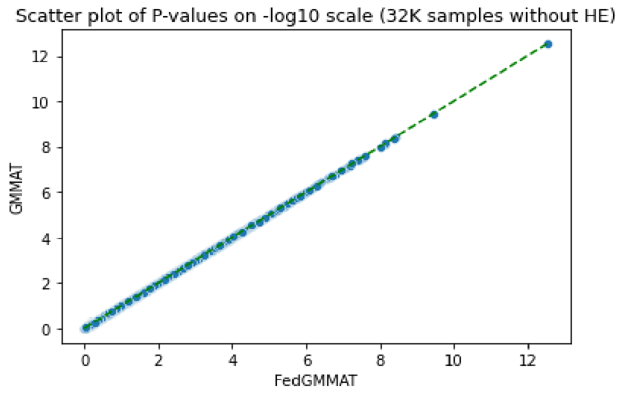

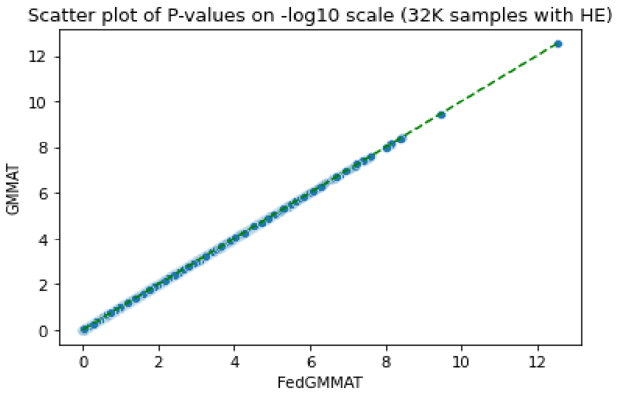

3. The following prior work on federated mixed model association tests should be discussed and compared to the proposed method:

<u>Authors Response:</u> We discuss and compare below each method to FedGMMAT:

- Yan, Z., Zachrison, K. S., Schwamm, L. H., Estrada, J. J., & Duan, R. (2023). A privacy-preserving and computation-efficient federated algorithm for generalized linear mixed models to analyze correlated electronic health records data. PloS one, 18(1), e0280192.

This method is for analyzing EHR datasets with a focus on building smaller scale models. We could not immediately identify a way to adopt this method for performing a GWAS using the additive mixed effect models phenotype used in statistical genetics.

- Li, W., Chen, H., Jiang, X., & Harmanci, A. (2023). Federated generalized linear mixed models for collaborative genome-wide association studies. Iscience, 26(8).

Please note that this is our previous method (dMEGA) to perform GWAS on a much smaller scale (several thousand variants), without a specific focus on using a single random intercept parameter. In comparison, FedGMMAT represents an important step forward in a more realistic setting for modeling phenotype using the additive mixed effects. In particular, dMEGA cannot readily model polygenic effects and has more computational requirements compared to FedGMMAT.

- Chen, J., Edupalli, M., Berger, B., & Cho, H. (2022). Secure and federated linear mixed model association tests. bioRxiv, 2022-05.

To our understanding, this method provides a federated approach for the application of linear mixed models. These approaches are effective for modeling continuous phenotypes. However, for the binary case/control studies, these linear mixed models may introduce biases since they do not capture the heteroskedasticity of the binary phenotype (i.e., variance-mean relationship) as demonstrated by our co-author Dr. Han Chen. Indeed, GMMAT method was specifically designed and developed to mitigate these limitations of the linear mixed models in binary traits and we are focusing on this problem for this specific purpose in developing FedGMMAT. Secondly, to our understanding, Chen-Edupalli et al.'s method can only handle random effects that stem from familial relationships, i.e., polygenic effects quantified by dense genetic relatedness matrix. It does not immediately provide the means of integrating other relationship information (e.g., shared environmental factors, longitudinal study designs).

On the other hand, we also would like to point out that, to our understanding, Chen-Edupalli et al.'s approach does not require explicit calculation of the GRM matrix and is more advantageous compared to FedGMMAT from this perspective. However, the approaches that do not explicitly calculate GRM matrix may have limitations when admixed populations exist in the cohorts[4]. Overall, both FedGMMAT and Chen et al's method provide their own set of advantages and we believe they are important contributions to the literature that different use cases can benefit from.

- Zhu, R., Jiang, C., Wang, X., Wang, S., Zheng, H., & Tang, H. (2020). Privacy-preserving construction of generalized linear mixed model for biomedical computation. Bioinformatics, 36(Supplement_1), i128-i135.
This method by Zhu et al. is one of the first methods to perform federated generalized mixed effects testing. We have extensively tested this algorithm in our previous study (dMEGA) and found that it does not report p-values for the genetic variants; it only reports the effect size estimates. Furthermore, it does not model the polygenic random effects caused by cryptic kinship. Finally, we also observed that Zhu et al.'s method exhibited poor convergence due to utilizing an MCMC-based approach.

Updates to Manuscript: We updated the results section with a new subsection named "Comparison to other Methods" and included the details of the methodological differences.

Minor comments:
1. The manuscript has many grammatical errors. The authors need to thoroughly revise the writing.
Response: In this revision, we went through the whole manuscript and fixed grammatical and writing errors, transitional breaks, and connections among the thought flow of the manuscript. We believe that the manuscript is much more readable. We thank the reviewer again for patience in evaluating the manuscript in its original form.

2. While it is emphasized that "null model fitting does not utilize any of the sensitive genotype data," it is important to note that the covariate and phenotype data are also sensitive.
Response: We agree with the reviewer and we updated this sentence and included the list of intermediate information that is shared with the central server to clarify the privacy concerns and limitations as a result of using FedGMMAT.

3. Round-robin updates appear less efficient compared with the approach of calculating all updates simultaneously and aggregating them to a single site.
Response: This is a great point. The aggregations can indeed be performed at one site by all sites sending to the aggregation site and the aggregation site submitting the encrypted aggregation to the central server. This would make the whole algorithm much more efficient since all sites can

send at the same time. In fact, this would most likely decrease the computation time by one-third for the large-scale tests. We have included this as a future design update to FedGMMAT.

4. Section IV-C (Parameters estimation) is difficult to follow as it lists the terms to be computed without providing explanations for each.

Response: We rephrased and explained these terms again to make it easier to follow. We hope that they are clearer. We sincerely thank the reviewer for their patience and careful evaluation.

5. To assess FedGMMAT's correction for kinship, it would be beneficial to include results from non-GMM association tests for comparison.

Response: We understand that this stems from our failure to motivate the importance of using GLMM for association testing, and we believe it is important to address this concern. To demonstrate the importance of GLMMs, we performed tests where we ran GMMAT and plink2 (non-mixed effects model method) on simulated datasets with high levels of heterogeneity in relatedness among the case/control cohorts. We simulated genotype data for 6500 variants for 4800 subjects from the GBR population in The 1000 Genomes Project. Among these, 2400 subjects were simulated as outbred individuals. Remaining 2400 subjects were simulated from 100 pedigrees of 24 subjects with moderate levels of inbreeding. The phenotypes were simulated with no genetic component: The inbred population is specifically set as the case population as the subjects with disease and the outbred population is set as the control population. For each subject, 5 PCs and a randomly assigned gender was used as fixed covariates.

We tested plink2 and GMMAT on these simulated datasets. Specifically, GMMAT was tested with kinship matrix inferred using SIGFRIED. As a negative control, we tested the GMMAT with a trivial kinship matrix, i.e., diagonal kinship matrix to simulate the lack of kinship information (Fig S1, copied below as well). We observed that there is strong inflation of p-values in plink2's p-values. As expected, GMMAT exhibits the same inflation of the p-values when it is run with the trivial (i.e., diagonal) kinship matrix. When GMMAT is run with the inferred kinship matrix, we observed that the biases due to kinship heterogeneity are accounted by GMMAT.

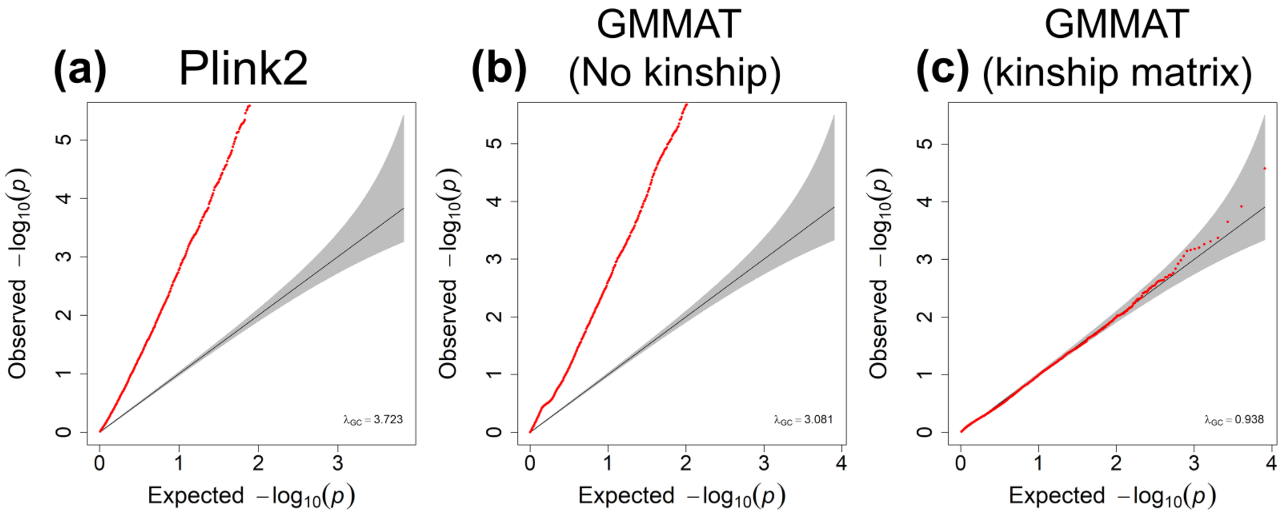

Overall, this result demonstrates an example scenario where the kinship heterogeneity among cases and controls may bias the results when cryptic kinship exists more in the cases or controls by random chance. We do acknowledge that this is a severe case of cryptic relatedness, and it is aimed at demonstrating the importance of considering polygenic effects caused by kinship. We also acknowledge that there can be different cases where cryptic kinship can bias the results[6].

REFERENCES:

1. Habier D, Fernando RL, Dekkers JCM. The impact of genetic relationship information on genome-assisted breeding values. Genetics. 2007;177: 2389–2397.

2. Harmanci AO, Kim M, Wang S, Li W, Song Y, Lauter KE, et al. Open Imputation Server provides secure Imputation services with provable genomic privacy. bioRxiv. 2021; 2021.09.30.462262.

3. Zhang Q-X, Liu T, Guo X, Zhen J, Yang M-Y, Khederzadeh S, et al. Searching across-cohort relatives in 54,092 GWAS samples via encrypted genotype regression. PLoS Genet. 2024;20: e1011037.

4. Tan T, Atkinson EG. Strategies for the genomic analysis of admixed populations. Annu Rev Biomed Data Sci. 2023;6: 105–127.

5. Wang S, Kim M, Li W, Jiang X, Chen H, Harmanci A. Privacy-aware estimation of relatedness in admixed populations. Brief Bioinform. 2022;23. doi:10.1093/bib/bbac473

6. Voight BF, Pritchard JK. Confounding from cryptic relatedness in case-control association studies. PLoS Genet. 2005;1: e32.
